# Delays in receiving obstetric care and poor maternal outcomes: results from a national multicentre cross-sectional study

**DOI:** 10.1186/1471-2393-14-159

**Published:** 2014-05-05

**Authors:** Rodolfo C Pacagnella, José G Cecatti, Mary A Parpinelli, Maria H Sousa, Samira M Haddad, Maria L Costa, João P Souza, Robert C Pattinson

**Affiliations:** 1 Department of Obstetrics and Gynaecology, School of Medical Sciences, University of Campinas, Campinas, Brazil; 2 Centre for Studies in Reproductive Health of Campinas (Cemicamp), Campinas, Brazil; 3 MRC Maternal and Infant Health Care Strategies Research Unit, University of Pretoria, Gauteng, South Africa

**Keywords:** Severe maternal morbidity, Maternal mortality, Maternal near miss, Delays in obstetric care, Emergency obstetric care

## Abstract

**Background:**

The vast majority of maternal deaths in low-and middle-income countries are preventable. Delay in obtaining access to appropriate health care is a fairly common problem which can be improved. The objective of this study was to explore the association between delay in providing obstetric health care and severe maternal morbidity/death.

**Methods:**

This was a multicentre cross-sectional study, involving 27 referral obstetric facilities in all Brazilian regions between 2009 and 2010. All women admitted to the hospital with a pregnancy-related cause were screened, searching for potentially life-threatening conditions (PLTC), maternal death (MD) and maternal near-miss (MNM) cases, according to the WHO criteria. Data on delays were collected by medical chart review and interview with the medical staff. The prevalence of the three different types of delays was estimated according to the level of care and outcome of the complication. For factors associated with any delay, the PR and 95%CI controlled for cluster design were estimated.

**Results:**

A total of 82,144 live births were screened, with 9,555 PLTC, MNM or MD cases prospectively identified. Overall, any type of delay was observed in 53.8% of cases; delay related to user factors was observed in 10.2%, 34.6% of delays were related to health service accessibility and 25.7% were related to quality of medical care. The occurrence of any delay was associated with increasing severity of maternal outcome: 52% in PLTC, 68.4% in MNM and 84.1% in MD.

**Conclusions:**

Although this was not a population-based study and the results could not be generalized, there was a very clear and significant association between frequency of delay and severity of outcome, suggesting that timely and proper management are related to survival.

## Background

Maternal mortality is a robust indicator of human development [1]. In many low- and middle-income countries, death rates related to pregnancy are often high and have an impact on reproductive-aged women. However, these deaths are mostly avoided by timely and adequate treatment [2]. Maternal mortality is an indicator of female status in society, considering access to health care and adequacy of the health system in responding to the needs of these women. Maternal death is closely associated with socioeconomic deprivations, which are difficult to alter. However, the prevention of maternal mortality is extremely sensitive to obstetric care standards and these can be modified [3].

The differences in maternal mortality ratios between low and high-income countries arise from management of pregnancy complications that can potentially lead to death. Around 99% of all maternal deaths occur in low- and middle-income countries. These deaths are mainly attributed to direct obstetric causes (haemorrhage, sepsis, complications of abortion and hypertensive disorders,) [4].

To improve medical care in obstetric emergencies, appropriate timing is extremely important [1]. Providing timely treatment for obstetric emergencies is the key to reduce maternal mortality [4]. Thaddeus and Maine [2] developed a three delays model two decades ago to evaluate the circumstances surrounding access to appropriate emergency obstetric care. Those authors described delay as having three components or phases: phase I – delay in deciding to seek care by the individual and/or family, phase II – delay in reaching an adequate health care facility, and phase III – delay in receiving adequate care at the health facility.

Since then, this “three delays model” has been very useful for the recognition and study of maternal mortality from the onset of complications. However, studies using death as the outcome usually face a challenge regarding the low absolute number of events [4]. This difficulty has been overcome since the 1990s by the study of a group of patients known as “maternal near miss” cases. These women, who escaped death by a stroke of luck or by receiving timely appropriate care after a severe complication during pregnancy, constitute a proxy for maternal death. In addition, these patients can provide direct information after the event. The discussion and evaluation of these cases is more readily accepted than maternal deaths by health professionals and institutions [5-7].

Recently, some authors have investigated delay related to a maternal near miss event, [5,7-13] although few have provided an analytical approach and most have encountered methodological problems [12]. The fact is that women die or at best almost die from preventable causes during pregnancy and childbirth. The reason is because life-threatening conditions may develop without warning and need to be promptly recognized and treated.

However, there is still a need to understand why and under which situation this condition occurs. Both the “demand side” of the barrier and the “supply” or “provider side” of the barrier may play an important role in outcome. Although intuitive, a direct association between the occurrence of delay and severity of maternal outcome has never been systematically demonstrated. Therefore, the aim of this study was to identify the occurrence of delay in providing health care among pregnant women with severe maternal morbidity in Brazil.

## Methods

### Design and setting

This study is part of the Brazilian Network for Surveillance of Severe Maternal Morbidity study, which is a cross-sectional multicentre study of 27 obstetric referral maternity hospitals in all geographical regions of Brazil, in a mix of health facilities (public and private health facilities, university and non-university hospitals) that provide specialized obstetric care and perform a minimum number of 1,000 deliveries per year. To be part of this study, the facility was required to have broadband internet connection, data on the prevalence of some obstetric interventions during delivery based on scientific evidence and the availability of written protocols of service procedures. The main goals of this network were to establish the prevalence of maternal near miss events among women admitted to these hospitals and prospectively evaluate the use of the new criteria for near miss events established by the World Health Organization in 2009 [14]. Methodological details related to the research protocol and its implementation are published elsewhere [15,16].

### Study size

We estimated that a sample of 390 cases of maternal near miss (MNM) would be sufficient to show a difference of 10% in a near miss incidence between adolescents and adults (α = 0.05, β = 0.2, the ratio between adolescents and adults was 4:1; the incidence of near miss between adolescents was 8.5/1000 live births and among non-adolescents it was 7.5/1000 live births). This was done because the aim of the original research was to also evaluate the occurrence of maternal morbidity specifically for adolescent mothers. Based on the prevalence of this condition in previous studies [17], a total of 75,000 births would have been monitored, therefore determining the number of health facilities taking part in the study.

### Procedures for subject selection and data collection

In each hospital, a local research team (with the local medical investigator plus a medical or nursing coordinator) performed a prospective surveillance on severe maternal morbidity, daily reviewing all women admitted to hospital, considering the inclusion criteria: the presence of at least one WHO potentially life-threatening condition (PLTC) (Table 1 – [14]). Data was collected between July 2009 and June 2010. During daily visits to the maternity wards, medical charts were selected for further data retrieval which was performed after hospital discharge, transfer to another hospital or death. If there was any missing information or doubt, the attending medical team was also contacted for necessary clarifications.

**Table 1:** **Potentially life-threatening maternal conditions **[14]

**Hemorrhagic complications**	**Hypertensive disorders**	**Other complications**	**Management indicators of severity**
Abruptio placentae	Severe preeclampsia	Pulmonary oedema	Transfusion of blood derivatives
Placenta previa/accreta/increta/percreta	Eclampsia	Seizures	Central venous access
Ectopic pregnancy	Hypertensive encephalopathy	Sepsis	ICU admission
Ruptured uterus	Severe hypertension	• Postpartum endometritis	Prolonged hospital stay (>7 days)
Severe hemorrhage due to abortion	HELLP syndrome	• Post abortion endometritis	Intubation unrelated to anaesthesia
Postpartum hemorrhage	Acute fatty liver of pregnancy	• Urinary infection	Return to operating room
• atony		• Chest infection	Major surgical intervention (hysterectomy, laparotomy)
• retained placenta		Thrombocytopenia <100 000	Use of magnesium sulphate
• Perineal lacerations		Thyroid crisis	
• Coagulopathy		Shock	
• Uterine inversion		Acute respiratory failure	
		Acidosis	
		Cardiopathy	
		Cerebrovascular accident	
		Coagulation disorders	
		Thromboembolism	
		Diabetic ketoacidosis	
		Jaundice/hepatic dysfunction	
		Meningitis	
		Acute renal failure	

Data was collected using an 80 item pre-coded form including data on patient demographic and economic characteristics, obstetric history, antenatal care status, previous morbid conditions, occurrence of life-threatening conditions and first complication in the chain of events leading to these conditions, duration of hospitalization, criteria for classification of severe maternal morbidity [14] maternal and neonatal outcomes, as well as information on the delay in providing care. Information on ethnicity/skin colour was also obtained from clinical records using the provider assignment. For data management we used an open-access, web-based database solution (OpenClinica®, Akaza Research, LLC, 2009, Waltham, MA, USA, https://community.openclinica.com/). This data management system is compliant with Good Clinical Practice (GCP) and regulatory guidelines, allowing differentiated user roles and privileges, password and electronic signatures, SSL encryption and de-identification of Protected Health Information (PHI).

### Financing and ethical aspects

The study was funded by the Department of Science and Technology from the Brazilian Ministry of Health and CNPq which played no other role in the development, data collection, analysis or interpretation of the results from this study. The study was approved by the National Council on Ethics in Human Research, by the local Institutional Review Boards of the coordinating centre (IRB from the School of Medical Sciences, University of Campinas - Approval letter CEP 097/2009) and also by local IRB of all the participating centres: Maternidade Cidade Nova Dona Nazarina Daou (Manaus, AM), Maternidade Climério de Oliveira (Salvador, BA), Hospital Geral de Fortaleza (Fortaleza, CE), Hospital Geral Dr. César Cals (Fortaleza, CE), Maternidade Escola Assis Chateaubriand (Fortaleza, CE), Hospital Materno Infantil de Goiania (Goiania, GO), Hospital Universitário da Universidade Federal do Maranhao (Sao Luis, MA), Maternidade Odete Valadares (Belo Horizonte, MG), Instituto de Saúde Elıdio de Almeida (Campina Grande, PB), Hospital Universitário Lauro Wanderley da Universidade Federal da Paraíba (Joao Pessoa, PB), Centro Integrado de Saúde Amaury de Medeiros (Recife, PE), Instituto de Medicina Integral Prof. Fernando Figueira (Recife, PE), Hospital das Clınicas da Universidade Federal de Pernambuco (Recife, PE), Hospital das Clınicas da Universidade Federal do Paraná (Curitiba, PR), Hospital Maternidade Fernando Magalhaes (Rio de Janeiro, RJ), Instituto Fernandes Figueira (Rio de Janeiro, RJ), Hospital das Clinicas da Universidade Federal do Rio Grande do Sul (Porto Alegre, RS), Faculdade de Medicina de Botucatu da Universidade Estadual Paulista (Botucatu, SP), Hospital da Mulher da Universidade Estadual de Campinas (Campinas, SP), Hospital e Maternidade Celso Pierro da Pontifícia Universidade Católica (Campinas, SP), Hospital Israelita Albert Einstein (São Paulo, SP), Faculdade de Medicina de Jundiaí (Jundiaí, SP), Hospital das Clınicas da Faculdade de Medicina de Ribeirão Preto da Universidade de São Paulo (Ribeirão Preto, SP), Santa Casa de Limeira (Limeira, SP), Santa Casa de São Carlos (São Carlos, SP), Casa Maternal Leonor Mendes de Barros (São Paulo, SP), Hospital São Paulo da Universidade Federal de São Paulo (São Paulo, SP). Approval included access to medical records of all women and their children.

### Participants

Women enrolled in the study were identified by the occurrence of any of the conditions listed in the WHO criteria - Table 1 as a potential life-threatening condition (PLTC). After hospital discharge and based on progression of the patient, each case was classified as a PLTC case, as a maternal near-miss (MNM) event or maternal death (MD), according to outcome [14]. By definition, a woman with a PLTC has a condition that could potentially lead to death due to haemorrhage, hypertension or other clinical and obstetrical complications or any indicators of severity. A MNM case was defined as “a woman who nearly died but survived a complication occurring during pregnancy, childbirth, or within 42 days of termination of pregnancy” [14]. This definition of maternal near miss includes clinical and laboratory evidence of organ dysfunction or failure, as well as any procedure for management that could be proxy of organ failure, such as intubation and ventilation unrelated to anaesthesia, any dose of continuous vasoactive drugs (dopamine, epinephrine or norepinephrine) required or hysterectomy for bleeding control.

### Main outcome measures

In all cases, we searched for data on quality of care indicators with possible shortcomings and delays that could cause or contribute to the occurrence of PLTC, MNM or MD. Since we were unable to identify the “real” delay in time from the onset of complication to outcome, we used the operational definition of “delay” as any shortcoming and failure at all levels of obstetric care that could led to a real delay in time.

These shortcomings/delays were classified as: a) sub-standard care/delay related to user factors – which refers to economic and educational status, a woman’s autonomy, illness-related behaviour, knowledge and attitudes about use of the health system and includes delay in identifying the condition, seeking medical care and refusing to accept treatment offered; b) sub-standard care/delay related to service accessibility – distribution of services, distance, transportation and general costs which included cases with difficulties in obtaining medical supplies or equipment which may lead to substandard care; and c) sub-standard care/delay related to quality of medical care – scope of medical services, management and support systems included delays in determining the appropriate diagnosis and providing appropriate patient treatment.

The local research investigator and coordinator were instructed to pursue evidence of delay regarding users, health service and medical care. First, medical records were scrutinized by the local researchers for data on patient decision to seek care, time from the onset of the problem to arrival, the woman’s pilgrimage, timely diagnosis, medication and blood products provided for the medical condition informed, patient referral, improper management and refusal by the patient or family member to accept treatment. Since all facilities had written protocols according to Ministry of Health recommendations, the study coordinators considered that management was improper when there was an evident discrepancy between the protocol and patient management.

Furthermore, the local research coordinator was encouraged to retrieve information from the medical staff who was asked to help identify gaps in information when completing the research form to find more evidence on the sequence of care offered to each woman. Neither the women nor family members were interviewed.

Local researchers were requested to seek more information, detail and documented delays, whenever there were reports of the occurrence of delay in medical records or when there was a positive impression by the staff responsible. When any delay related to health service and medical care could be identified, the level of care (primary, secondary or tertiary) was further specified. All cases were reviewed by the coordinating centre.

### Data management

To minimize data collection bias and to ensure the high quality of information, standard procedures were adopted for all cases. These procedures included preparatory meetings, site visits, close monitoring of data collection and data entry, concurrent query management, inconsistency checks, double data collection for selected medical charts, and the use of a detailed manual of operation. During site visits, implementation of the study was assessed and randomly selected medical records were checked against data already included in the database [15].

Implementation of auditing to record/monitor access and changes in data aligned with a set of validation/cross-checking rules was part of online data management. Checking rules related to delays were considered as follows:

• When there was “absent antenatal care,” the researchers considered delay related to health service accessibility. Although in Brazil there are generally good antenatal healthcare services available, there is a major difference between services available in diverse regions of the country, due to institutional problems, i.e. insufficient number of health professionals and the low quality of health services. Thus, it is assumed that inadequate antenatal care is an institutional problem more than an individual decision. In addition, if the number of antenatal visits was below the minimum for that specific gestational age, as recommended by the Brazilian Ministry of Health, antenatal care was considered “inadequate”.

• When “direct inter-hospital transfer” was checked in the form, researchers considered delay related to health service accessibility. In Brazil, the transfer of a pregnant woman to a tertiary referral hospital is theoretically mediated by the assistance of a public call centre regulating service availability, checking for the number of existing beds in these units daily and deciding where to transfer each specific patient, according to geographical location and resources.

• In cases of severe preeclampsia/eclampsia, researchers looked for magnesium sulphate administration as a management criterion. For all patients who did not receive magnesium sulphate, local researchers were asked to identify the criterion used to classify preeclampsia as severe and the possibility of delay related to quality of medical care.

• When “discharge required by the patient” or “evasion” was identified, the delay was considered due to “refused treatment” and was related to user factors.

• We considered that when a legal base to support safe abortion is lacking, women usually waited longer to seek medical care after they suffered an abortion. Therefore, this was classified as related to user factors.

• Finally, all data entered into the form (open field) was individually evaluated by researchers at the coordinating centre. With the explanations provided, it was often possible to understand and set specific delays in care.

### Statistical analysis

Using this comprehensive package of data quality procedures, reliable and high quality information was obtained. Data was analysed by the principal investigators who were not involved in data collection using EpiInfo® and SPSS® software. Initially, the occurrence of all types of delays was described according to the level of care and maternal outcome. On bivariate analysis, χ^2^ or Fisher’s exact tests were used to compare groups controlled by the cluster design in the analysis. Missing data was excluded and the total number available for each analysis was shown in tables. To assess the role of selected socio-demographic, antenatal and obstetric variables as predictors of delays, the prevalence ratios (PR) and their respective 95% CI were estimated, also adjusted for cluster design effect. To identify factors independently associated with the occurrence of any delay, multivariate analysis was conducted (Poisson multiple regression model) using pseudo-maximum-likelihood estimates with stepwise backward elimination procedure, removing from the model variables with the largest p-value until no variable with p-value > 0.05 remained.

## Results

During a 12-month period, 82,144 live births were screened, prospectively identifying 9,555 (11.6%) of PLTC, MMN or MD cases. Information on delays was not available for 839 cases (748 for PLTC, 77 for MNM and 14 for MD, a mean of 8.7% of missing data). A total of 140 (0.17%) maternal deaths and 770 (0.94% of live births) near miss cases were reported during the period of data collection.

Any kind of delay was observed in 53.8% of all subjects. Delay related to health service accessibility occurred most frequently (34.6%), followed by delay related to quality of medical care (25.7%), while delay related to user factors was observed in 10.2% of the records. Most delays in health service accessibility were related to difficulties in obtaining antenatal care, since more than 30% of women were categorized as receiving absent or inadequate antenatal care (8.2% of all women had no antenatal care visit) (Table 1). Delay in seeking health care and refusing treatment was more frequent when related to user factors, while problems in antenatal care was the most prevalent component of delay in health service accessibility. For delay related to quality of care, the most important component was improper patient management, followed by difficulty in communicating with the regulatory centre and delay in starting treatment (Table 2).

**Table 2:** **Proportion of cases of obstetric complications** (**n** = **9555**) **with identifiable sub**-**standard care**/**delays in the Brazilian network for surveillance of severe maternal morbidity study**

Delays*	**n (%)**
**User factors**^**a**^	**853 (10.2)**
Delay in seeking health services^a^	442 (5.3)
Refuse to treatment^a^	426 (5.1)
Unsafe abortion^a^	51 (0.6)
**Health Service Accessibility**^**b**^	**2906 (34.6)**
Difficulty in gaining access to antenatal care^c^	126 (1.4)
Difficulties or problems with transportation city/hospital^c^	117 (1.3)
Absent or inadequate antenatal care^a^	2692 (32.1)
Geographical difficulty in gaining access to health service^a^	198 (2.4)
**Quality of medical care**^**d**^	**2309 (25.7)**
Absence of blood products^c^	57 (0.6)
Lack of medication: magnesium sulphate, antibiotics, vasoactive drugs, uterotonics^c^	117 (1.3)
Difficulty in communicating between hospital and regulatory centre^c^	779 (8.8)
Lack of trained staff^c^	271 (3.1)
Difficulty in monitoring^c^	409 (4.6)
Delay in case referral/transfer^e^	292 (3.3)
Delay in diagnosis^e^	487 (5.5)
Delay in starting treatment^e^	602 (6.7)
Improper patient management^e^	1218 (13.6)
**Any delay**^**f**^	**4687 (53.8)**

*categories are not mutually exclusive.

Number of cases with information available: a – 8380; b – 8391; c – 8848; d – 8986; e – 8931; f - 8716.

The quality of medical care related to delay was classified according to the role of the health facility (Table 3). Inappropriate patient management was the most prevalent delay identified (13.6%) that occurred most frequently at tertiary level (9.8%). Difficulties with communication between health facilities and the regulatory centre responsible for managing patient referral, was also prevalent and occurred mostly in secondary care, as well as all delays related to staff training and qualification. Lack of medication or equipment was less commonly observed. Nevertheless, it occurred most frequently at secondary care level.

**Table 3:** **Proportion of cases of obstetric complications** (**n** = **9555**) **with sub**-**standard care**/**delays related to quality of care according to the level of health facility**

		**Level of health service in which the delay was identified**		
**Delays**	**n (%)**	**Primary health care**	**Secondary health care**	**Tertiary health care**
**Quality of medical care**^d^*	**2309 (25.7)**			
Absence of blood products^c^	57 (0.6)	9 (0.1)	41 (0.5)	7 (0.1)
Lack of medication: magnesium sulphate, antibiotics, vasoactive drugs, uterotonic^c^	117 (1.3)	36 (0.4)	56 (0.6)	25 (0.3)
Difficulty in communicating between hospital and regulatory centre^c^	779 (8.8)	139 (1.6)	425 (4.8)	215 (2.4)
Lack of trained staff^c^	271 (3.1)	74 (0.8)	144 (1.6)	53 (0.6)
Difficulty in monitoring^c^	409 (4.6)	21 (0.2)	256 (2.9)	132 (1.5)
Delay in case referral/transfer^e^	292 (3.3)	61 (0.7)	179 (2.0)	52 (0.6)
Delay in diagnosis^e^	487 (5.5)	93 (1.0)	266 (3.0)	128 (1.4)
Delay in starting treatment^e^	602 (6.7)	79 (0.9)	315 (3.5)	208 (2.3)
Improper patient management^e^	1218 (13.6)	69 (0.8)	277 (3.1)	872 (9.8)

Number of cases with information available: c – 8848; d – 8986; e – 8931.

*the categories are not mutually exclusive.

**Figure 1:**
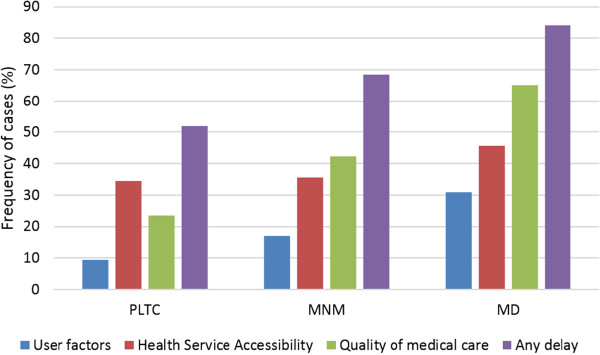
**Rates of delays in obtaining care among women with obstetric complications related to health services, health professionals or patients and their relatives according to maternal outcome (PLTC: potentially life threatening condition, MNM: maternal near miss, or MD: maternal death).** p-value < 0.001 except for Health Service Accessibility.

There was a positive association between the occurrence of any delay and severity of maternal outcome, as shown in Table 4. Any delay occurred in 84% of MD, in 68% of MNM and in 52% of PLTC cases, showing a significant increase in delay with severity of outcome. Figure 1 shows this trend. Delays related to user factors increased from 9.3% among PLTC subjects to 30.9% in MD, and delay in seeking health services was also related to outcome severity. All delays related to quality of medical care were also associated with a worse maternal outcome. However, “difficulties or problems with transportation” were the only delays related to health service accessibility significantly associated with the severity of outcome.

**Table 4:** **Proportion of identified cases of obstetric complications with sub**-**standard care**/**delay in receiving care according to the severity of maternal outcome**

**Delays**	**PLTC**	**MNM**	**MD**	p-value*
**Users’ factors**	709 (9.3)	110 (17.0)	34 (30.9)	**<0.001**
Delay in seeking health services	339 (4.4)	74 (11.4)	29 (26.4)	**<0.001**
Refuse to treatment	380 (5.0)	36 (5.6)	10 (9.1)	0.333
Unsafe abortion	41 (0.5)	8 (1.2)	2 (1.8)	0.285
**Total**	**7623**	**647**	**110**	
**Health Service Accessibility**	2622 (34.4)	232 (35.6)	52 (45.6)	0.078
**Total**	**7626**	**651**	**114**	
Difficulty in gaining access to antenatal care	106 (1.3)	16 (2.2)	4 (3.2)	0.191
Difficulties or problems with transportation city/ hospital	83 (1.0)	19 (2.6)	15 (11.9)	**<0.001**
Subtotal	8002	720	126	
Absent or inadequate antenatal care	2442 (32.0)	211 (32.6)	39 (35.5)	0.713
Geographical difficulty in gaining access to health service	172 (2.3)	22 (3.4)	4 (3.6)	0.072
Subtotal	7623	647	110	
**Quality of medical care**	1919 (23.6)	306 (42.3)	84 (65.1)	**<0.001**
**Total**	**8134**	**723**	**129**	
Absence of blood products	28 (0.3)	23 (3.2)	6 (4.8)	**<0.001**
Lack of medication: magnesium sulphate, antibiotics, vasoactive drugs, uterotonics	81 (1.0)	26 (3.6)	10 (7.9)	**<0.001**
Difficulty in communicating between hospital and regulatory centre	647 (8.1)	100 (13.9)	32 (25.4)	**0.005**
Lack of trained staff	189 (2.4)	59 (8.2)	23 (18.3)	**<0.001**
Difficulty in monitoring	270 (3.4)	105 (14.6)	34 (27.0)	**<0.001**
Subtotal	8002	720	126	
Delay in case referral/transfer	177 (2.2)	82 (11.4)	33 (26.4)	**<0.001**
Delay in diagnosis	340 (4.2)	113 (15.7)	34 (27.2)	**<0.001**
Delay in starting treatment	439 (5.4)	120 (16.7)	43 (34.4)	**<0.001**
Improper patient management	1029 (12.7)	145 (20.2)	44 (35.2)	**0.003**
Subtotal	8088	718	125	
**Any delay**	4107 (52.0)	474 (68.4)	106 (84.1)	**<0.001**
**Total**	**7897**	**693**	**126**	

*Chi-square test considering the cluster design. P-values in bold mean they are statistically significant.

Considering all PLTC, infections showed a high prevalence ratio for any delay (Table 5). The occurrence of any delay was significantly more prevalent among adolescent, non-white women, with a low schooling status and publicly funded hospitalization (Table 6). When antenatal care was performed at the same facility and privately sponsored, there was a significantly lower prevalence of any delay. In addition, if any transfer was necessary for women having access to the facility, the risk of any delay was also higher (Table 7).

**Table 5:** Estimated risks of any delay in obstetric care according to PLTC diagnosis

	**Any delay (%)**				
**Diagnosis leading to PLTC**	**Yes**	**No**	**p-value**	**PR**	**95% CI**
Hemorrhage	23.1	24.8	0.639	0.93	0.70-1.24
Hypertension	72.3	67.2	0.135	1.07	0.95-1.21
Infection	**1.5**	**0.6**	**0.005**	**2.54**	**1.33-4.84**
Other Clinical conditions	10.1	11.5	0.382	0.87	0.65-1.18

n = 8716. Values in bold mean they are statistically significant. P-values in bold mean they are statistically significant.

**Table 6:** **Estimated risks of any sub**-**standard care**/**delay in obstetric care according to some socio**-**demographic characteristics**

**Characteristics**	**User factors**	**Health service accessibility**	**Quality of medical care**	**Any delay (%)**		**PR**_**adj**_	**CI 95%**
	**%**	**%**	**%**	**Yes**	**No**		
**Age**							
< 19	15.6	19.9	18.3	19.1	16.3	**1.09**	**1.02**-**1.15**
20-29	50.3	47.2	47.5	47.3	48.4	ref	
30-39	28.5	27.5	29.8	28.5	30.7	0.98	0.92-1.03
≥40	5.6	5.3	4.5	5.1	4.6	1.06	0.96-1.16
Total (n)	(853)	(2906)	(2309)	(4687)	(4029)		
p-value*	0.395	**<0.001**	0.758	**0.008**			
**Maternal ethnicity**							
White	39.0	36.6	36.2	38.1	48.0	Ref	
Non-white	61.0	63.4	63.8	61.9	52.0	**1.22**	**1.07**-**1.39**
Total (n)	(671)	(2258)	(1716)	(3550)	(3273)		
p-value*	0.514	**0.002**	0.204	**0.006**			
**Educational level**							
Basic	51.1	54.2	45.0	50.7	42.5	**1.45**	**1.15**-**1.84**
High-school	45.3	42.6	48.9	44.9	50.0	**1.27**	**1.04**-**1.56**
College	3.6	3.1	6.1	4.3	7.5	ref	
Total (n)	(591)	(2289)	(1551)	(3415)	(3087)		
p-value*	0.141	**<0.001**	0.804	**<0.001**			
**Marital status**							
Cohabitating	51.6	48.3	57.2	52.1	55.7	ref	
No cohabitating	48.4	51.7	42.8	47.9	44.3	1.07	0.95-1.21
Total (n)	(703)	(2503)	(1846)	(3903)	(3578)		
p-value*	0.491	**0.003**	0.433	0.242			
**Insurance for hospitalization**							
Public	99.5	99.8	99.2	99.4	98.1	**1.98**	**1.40**-**2.79**
Private/Insurance	0.5	0.2	0.8	0.6	1.9	ref	
Total (n)	(853)	(2902)	(2307)	(4682)	(4026)		
p-value*	**0.027**	**<0.001**	0.139	**<0.001**			

*Chi-square test considering the cluster design PR_adj_: Prevalence Ratio adjusted for cluster effect.

Values in bold mean they are statistically significant.

**Table 7:** **Estimated risks of any sub**-**standard care**/**delay in obstetric care according to some characteristics of antenatal care**

**Characteristics**	**User factors**	**Health service accessibility**	**Quality of medical care**	**Any delay (%)**		**PR**_**adj**_	**CI 95%**
	**%**	**%**	**%**	**Yes**	**No**		
**Antenatal care in the same facility**							
Yes	20.0	20.4	15.4	18.9	28.3	ref	
No	67.0	70.0	80.2	73.6	68.3	**1.28**	**1.06-1.53**
No antenatal care	13.0	9.6	4.4	7.5	3.4	**1.66**	**1.26-2.19**
Total (n)	(769)	(2656)	(2045)	(4212)	(3692)		
p-value	**<0.001**	**<0.001**	0.058	**0.002**			
**Antenatal insurance**							
Public	96.3	96.6	94.5	95.9	93.4	**1.31**	**1.03-1.67**
Private/Insurance	3.7	3.4	5.5	4.1	6.6	ref	
Total (n)	(588)	(2174)	(1703)	(3426)	(3227)		
p-value	0.079	**0.012**	0.789	**0.010**			
**Access to the hospital**							
Spontaneous demand	50.1	48.2	29.3	42.0	55.2	ref	
Transfer from Emergency Department	2.6	2.5	2.3	2.2	1.2	**1.45**	**1.18-1.78**
Scheduled Inter-hospital transfer	21.3	22.9	20.8	22.2	18.7	**1.24**	**1.03-1.49**
Direct Inter-hospital transfer	6.4	8.5	29.8	14.8	0.6	**2.08**	**1.73-2.50**
Referral from another Health Service	11.8	9.4	11.1	10.6	13.1	1.03	0.87-1.22
Referral from the same Health Service	8.0	8.6	6.7	8.1	11.2	0.97	0.88-1.07
Total	(817)	(2759)	(2149)	(4401)	(3898)		
p-value	0.685	**0.014**	**<0.001**	**<0.001**			

PR_adj_: Prevalence Ratio adjusted for cluster effect. Values in bold mean they are statistically significant.

Table 8 shows the obstetric characteristics. Multiparity, lower gestational ages on admission and pregnancy termination, postpartum admission, induced and unsafe abortion were associated with delays. Some women were admitted in the postpartum or post abortion period and gestational age could not be defined. The “still pregnant” option refers to women who remained pregnant at the end of data collection for the case (either due to hospital discharge or hospital transfer). There was no difference in frequency of delays with respect to mode of delivery and presence of pre-existing health problems. On multiple analysis (Table 9), any delay was independently associated with publicly sponsored hospitalization, gestational age below term at pregnancy termination or still pregnant, non-white woman, antenatal care at another health facility, previous abortions, low schooling and public insurance for prenatal care.

**Table 8:** **Estimated risks of any sub**-**standard care**/**delay in obstetric care according to some obstetric characteristics**

**Characteristics**	**User factors**	**Health service accessibility**	**Quality of medical care**	**Any delay (%)**		**PR**_**adj**_	**CI 95%**
	**%**	**%**	**%**	**Yes**	**No**		
**Number of gestations**							
1	34.9	38.4	43.6	40.6	42.8	ref	
2 to 3	37.6	36.6	37.3	36.8	40.4	0.98	0.93-1.04
≥4	27.5	25.1	19.0	22.6	16.9	**1.16**	**1.06-1.28**
Total (n)	(845)	(2897)	(2290)	(4658)	(4021)		
p-value	**<0.001**	**<0.001**	0.289	**<0.001**			
**Number of births**							
0	40.9	43.7	49.9	46.3	49.7	ref	
1 to 2	39.4	37.8	36.9	37.6	39.8	1.01	0.95-1.06
≥3	19.6	18.6	13.2	16.1	10.5	**1.23**	**1.10-1.38**
Total (n)	(845)	(2897)	(2290)	(4658)	(4021)		
p-value	**0.002**	**<0.001**	0.235	**<0.001**			
**Number of previous C-sections**							
0	72.7	73.6	76.1	74.6	76.7	ref	
1	18.5	18.0	17.0	17.4	16.9	1.03	0.96-1.09
≥2	8.8	8.4	6.9	7.9	6.4	1.11	1.00-1.24
Total (n)	(816)	(2848)	(2227)	(4570)	(4003)		
p-value	0.129	**0.017**	0.806	0.065			
**Number of abortions**							
0	71.4	76.9	78.4	76.9	78.2	ref	
≥1	28.6	23.1	21.6	23.1	21.8	1.04	0.97-1.10
Total (n)	(844)	(2897)	(2290)	(4657)	(4020)		
p-value	**0.002**	0.517	0.510	0.268			
**Gestational age on admission**							
<22	13.0	5.0	5.2	5.5	5.9	1.10	0.88-1.39
22 a 27	7.9	6.1	6.5	6.2	5.0	**1.27**	**1.05-1.52**
28 a 33	20.5	20.4	24.5	21.5	16.1	**1.30**	**1.12-1.51**
34 a 36	18.9	21.8	21.5	21.4	17.4	**1.26**	**1.11-1.42**
≥37	31.8	43.5	33.4	39.4	52.1	ref	
postpartum/post-abortion	7.8	3.2	9.0	6.0	3.4	**1.43**	**1.14-1.80**
Total	(808)	(2839)	(2257)	(4558)	(3924)		
p-value	**<0.001**	**0.025**	**<0.001**	**<0.001**			
**Gestational age on pregnancy termination**							
<22	10.8	3.1	4.0	3.7	3.4	1.16	0.95-1.41
22 to 27	3.9	3.2	3.4	3.3	2.7	**1.23**	**1.03-1.46**
28 to 33	16.9	16.7	21.4	17.6	12.5	**1.29**	**1.12-1.49**
34 to 36	18.6	21.4	23.5	22.0	16.5	**1.27**	**1.13-1.43**
≥37	37.9	48.5	40.7	45.8	57.5	ref	
postpartum/post-abortion	11.8	7.2	7.0	7.6	7.4	1.13	0.91-1.40
Total	(762)	(2800)	(2139)	(4408)	(3840)		
p-value	**<0.001**	0.111	**<0.002**	**<0.001**			
**Onset of abortion**							
Spontaneous	44.0	50.0	59.5	51.7	74.4	ref	
Induced	56.0	50.0	40.5	48.3	25.6	**1.47**	**1.13-1.91**
Total (n)	(75)	(54)	(42)	(116)	(82)		
p-value	**<0.001**	0.069	0.742	**<0.002**			
**Safety of Abortion**							
Safe	35.2	50.9	73.0	55.7	96.3	ref	
Unsafe	64.8	49.1	27.0	44.3	3.7	**2.18**	**1.64-2.91**
Total (n)	(71)	(53)	(37)	(106)	(81)		
p-value	**<0.001**	**0.010**	0.934	**<0.001**			
**Pre-existing health conditions**							
Yes	53.7	49.9	47.1	49.0	49.3	0.99	0.88-1.13
No	46.3	50.1	52.9	51.0	50.7	ref	
Total (n)	(752)	(2609)	(1857)	(4019)	(3815)		
p-value	0.182	0.509	0.717	0.928			
**Mode of delivery**							
Vaginal	16.2	25.5	17.7	21.8	24.4	ref	
C-section	57.9	63.8	69.9	65.7	62.4	1.08	0.96-1.22
Abortion/ectopic	15.3	3.7	5.9	5.4	6.0	1.00	0.75-1.32
Still pregnant	10.6	7.0	6.6	7.2	7.1	1.06	0.84-1.34
Total (n)	(850)	(2901)	(2299)	(4674)	(4018)		
p-value	**<0.001**	0.050	0.142	0.538			

PR_adj_: Prevalence Ratio adjusted for cluster effect. Values in bold mean they are statistically significant.

**Table 9:** **Factors independently associated with any sub**-**standard care**/**delay in receiving care identified among women with obstetric complication** (multiple analysis by Poisson regression*; **n** = **4**,**794**)

**Variable**	**PR**_**adj**_	95% CI*	**P**
Insurance for hospitalization (Public)	1.96	1.47–2.60	<0.001
Gestational age at pregnancy termination (postpartum/ post-abortion or <37 weeks)	1.32	1.13–1.55	<0.002
Ethnicity (Non-white)	1.26	1.10–1.46	0.002
Antenatal care at the same facility (No/no PN)	1.30	1.10–1.53	0.003
Number of previous abortions (≥1)	1.07	1.02–1.13	0.011
Schooling (up to high school)	1.27	1.03–1.57	0.025
Insurance for prenatal care (Public)	0.87	0.76–0.99	0.043

PR_adj_: Prevalence ratio adjusted for cluster design; *Analysis considering the cluster design (centre).

Predictors included in the multivariable model: age, ethnicity, educational level, marital status, insurance for hospitalization, antenatal care at the same facility, insurance for antenatal care, way of access to hospital, number of pregnancies, number of previous births, number of previous C-sections, number of abortions, gestational age at admission, gestational age at pregnancy termination, pre-existing health conditions and mode of delivery.

## Discussion

Considering the occurrence of delay in providing health care to pregnant women, there was a positive association between delay in obstetric care and severity of adverse maternal outcomes. Overall, delay was identified in almost 54% of all cases. There were 52% with at least one delay among women with PLTC, 68% in MNM group and 84% in MD group. Although these figures seem to be well anticipated, to the best of our knowledge this is one of the few studies to address the occurrence of delay in obstetric care among women presenting with severe maternal morbidity. This is also the first time that a gradient of considerable delay associated with severe maternal outcome has been demonstrated. Other studies have analysed only maternal deaths or near miss cases, but current data enabled us to compare delays observed in groups with extremely bad outcome and a group of women with less severe conditions.

The main assumption behind the near miss approach is that both conditions share specific characteristics related to organ failure. Therefore, a woman experiencing a near miss event may be used as proxy for maternal death [18-21]. However, there is actually a difference between both conditions that can be associated with individual, as well as management aspects. Some of our findings show that patient management may play an important role in this process.

Many authors have found associations between delay in obstetric care and maternal outcomes, mainly MD [10,22]. More recently, studies of MNM outcome have also addressed information on delays [5,7-10,12]. Substandard care and delay Phase I could be identified in more than half of the cases of severe maternal morbidity in an audit study [5]. Reviewing clinical and administrative data, another Brazilian study identified some delay in 34% of MD and MNM cases [11] and delay in receiving care was found in 20%. Seeking care took longer than expected in 14% and delay in reaching care was found in 4%. Another study using maternal near-miss audit surveys found 9% of women with more than one delay in reaching the referral facility [13]. Studies of MNM found that the majority of patients arrived at the health facility in an extremely severe clinical condition, suggesting that women need to overcome certain obstacles to reach adequate care [7,12,23,24].

In our study, delays related to user factors (patients and/or their family) were less observed than others. However, although phase one delay is a matter of importance in many settings [7,10,13,25], in the present study there was no association between most types of delay and maternal outcomes, except for delay in seeking health services, which was 2.5 times more frequent in MNM and increased 6-fold in the MD group in comparison to the PLTC group. Lack of association may result from a potential weakness of the study regarding the assessment of the first delay (or shortcoming): medical records may not be the most reliable source of information. However, despite not having interviewed the women or family member, research coordinators and investigators were stimulated to carefully audit medical records soon after hospital discharge and to discuss any suspicion of delay with the attending medical and nursing staff, which could improve the identification of such delay.

The first delay is a relatively common finding in the literature. According to Thaddeus and Maine [2] phase 1 delay is often discussed as a barrier or constraint to the use of health services, a process dependent on a sociocultural and economic context, resulting from the interaction between infrastructure, distance from maternal health facilities, the cost of maternal care, and quality of care [2,26]. There is some evidence that time to seek care differs widely among complications [12]. Women may seek care only after recognizing that their condition is potentially life-threatening, even though there are studies suggesting that these women may be unable to make such judgment [5,10,25-27]. Evidence also suggests that providers do not attempt to adequately explain to women how to recognize severe obstetric problems and how to deal with them [22]. There is a need to educate the community for early recognition of symptoms to support timely decisions [25]. This is one purpose of antenatal care.

Inadequacy of antenatal care was the most frequently identified factor, present in more than 30% of cases. Antenatal care plays a very important role in promoting obstetric care, since the quality of antenatal care is a determinant of health education, facilitating the use of emergency obstetric care [28,29]. In Brazil, the use of antenatal services is high. Less than 3% of all pregnant women currently deliver without a single antenatal care visit [30]. However, this is not valid for all Brazilian regions. The indicator hides important differences between regions of the country. In 2010, the Southern region showed 75.3% of live births with 7 or more antenatal care visits, while in the North the proportion was 36.8% [31]. Data of the current study shows that difficulties and problems in patient referral and transfer are major barriers to the achievement of adequate emergency obstetric care. These problems occur mostly at secondary care level, which is the least developed level in Brazil. It is estimated that 15% of all pregnant women will develop pregnancy-related complications requiring access to a higher level of care [32]. Some studies found that a significant proportion of MNM or MD cases were already in critical clinical condition when reaching the facility [7,8,13,33-35] and important delays were found in such cases.

Our data also showed delay in reaching the referral hospital, which suggests referral system deficiencies. Barriers encountered by women seeking care, compromise timely access to obstetric emergency care, once a complication is recognized [34]. Although institutional births in Brazil account for almost one hundred percent of all deliveries [36] and maternal deaths are rare in the community, arriving at an adequate facility in time of dire need still seems to be a problem in our country.

The most important delay identified was related to the quality of medical care. It was most commonly observed at tertiary level. Delay related to medical staff is a key point in maternal care. It has been shown that delay by the professional is the most substantial contributor to substandard care even in high-income settings [37]. Even in low resource settings this is a very important issue to be considered: a recent systematic review on phase III delay found that it made a significantly greater contribution to maternal mortality than others [38].

The problem occurs in initiating adequate treatment when the woman has already reached the health care service [11]. The reasons for delay usually include costs leading to shortage of supplies, blood products and lack of technical competence among staff and poor attitude towards the patient [5,22,39]. However, these are multiple and complex causes. According to a systematic review, inadequate training/skills, drug procurement/logistics problems, staff shortage, lack of equipment and low staff motivation are the most commonly cited barriers at this level of care [38]. In many hospitals and within different contexts there are no emergency drugs immediately available in health services, increasing the burden of an obstetric complication [8].

Furthermore, the poor quality of care in tertiary facilities [40,41] contributes to maternal mortality, both directly (suboptimal standard of emergency care) and indirectly (poor quality of services) discourage women from seeking care [42]. Successful treatment of physical illness often requires the management of cognitive, psychological, and social factors [43]. Women experiencing near-miss episodes clearly show issues related to perceived quality of care [7] and this should be a matter of concern to health care facilities.

Emergency Obstetric Care (EmOC) facilities usually have a huge case load with severe conditions. There is no clear policy on adequate treatment of life-threatening emergencies [44]. To overcome this difficulty many strategies proved to be suitable such as audit and feedback. The study has some limitations that should be considered during the interpretation of the results. First, we were unable to directly measure the delay in time from onset of complication to outcome. Therefore, we measured proxy indicators that may lead to delays. We were not allowed to explore one of the most interesting characteristics of near miss cases: talking to women after occurrence of the event at a time when more information was available [7,8,28]. As a result, we were unable to address qualitative information on the cause and time of delay. In addition, some information gathered was based on provider assignment. For example, ethnicity proved to be independently associated with any delay and did not allow a broad comprehension of this relationship. The topic is of interest and will be specifically focused on another in-depth analysis. Although the study was performed in all geopolitical regions of Brazil, this was not a population-based study and it did not intend to represent all births occurring during data collection. Therefore, the results cannot be generalized to the Brazilian population. Nevertheless, this study may be representative of all pregnant women who obtained access to the health system and gave birth in tertiary hospitals during the study period.

In addition, in most facilities the research coordinator and investigator were part of the staff. This could generate a bias because these professionals could avoid recognizing a delay due to improper patient care. In low- and middle-income countries, the accuracy of medical records in capturing clinical activities and outcome is usually poor. When we consider such a sensitive issue as medical error, this information may be even less reliable [45] Reporting medical deficiencies and errors possibly face some organizational and cultural barriers (inevitability of error, habit, collegial bond), that could lead to bias in gathering information [45-49]. However, we believe that since a near miss event represents a positive outcome, in contrast to maternal death, some barriers in reporting medical and institutional errors may have been even weaker [50].

When considering that delay in providing proper patient treatment may reflect medical or institutional error, this information could have been even less reported than what we found. However, as presented in the methods section, we performed a rigid process of consistency checking which allowed data to be less prone to subjectivity among different investigators.

Finally, since we collected data on different outcomes, we were able to provide a risk estimate for delays. This analytical approach and the large sample size gave the present study the power to deal with the topic. To the best of our knowledge, this is the first time this data has been prospectively and systematically collected using the WHO definition and criteria [14] which can allow further comparison.

Results related to accessibility to health service and quality of care need to be highlighted, since they may have an impact on health policies. In Brazil, and in other countries where access to health system is similarly improving, public policies in maternal health should address the need for prompt access to an adequate obstetric care facility. In a more aggressive way, efforts and resources need to be focused on strengthening the ability of the health system and health professionals to deal with maternal complications. Therefore, the results of the present study must be seen through the perspective of quality of care assessment. Most importantly, there is a need to implement reporting systems of near miss events and delays related to these events that could allocate resources to correct the gaps in obstetric care [50,51].

## Conclusion

This study found a clear association between the occurrence of delay in gaining access to obstetric care and maternal deaths/maternal near misses. Furthermore, we identified an increasing frequency of delay as the outcome became worse, suggesting that despite the expected prevalence of clinical complications in pregnancy, the difference between life and death in obstetrics may be a matter of timely and proper management.

## Abbreviations

EmOC: Emergency Obstetric Care; MD: Maternal death; MNM: Maternal near-miss; MSI: Maternal Severity Index; PLTC: Potentially life-threatening conditions.

## Competing interests

The authors declare that they have no conflict of interests.

## Authors’ contributions

The idea for the study and this specific analytic approach arose in a group discussion among all the authors. Analyses were planned and performed by RCP, JGC and MHS. The first version of the manuscript was drafted by RCP, and then complemented with suggestions made by all the other authors. JGC supervised the entire process. All authors contributed to the development of the study protocol and approved the final version of the manuscript.

## Pre-publication history

The pre-publication history for this paper can be accessed here:

http://www.biomedcentral.com/1471-2393/14/159/prepub

## National network for the surveillance of severe maternal morbidity group **(**Email: jgcecatti@terra.com.br**)**

Fernanda G. Surita, João L. Pinto e Silva, Rodrigo S. Camargo, Vilma Zotareli, Lúcio T. Gurgel, Lale Say, Marilza V Rudge, Iracema M Calderon, Maria V Bahamondes, Danielly S Santana, Simone P Gonçalves, Eliana M Amaral, Olímpio B Moraes Filho, Simone A Carvalho, Francisco E Feitosa, George N Chaves, Ione R Brum, Gloria C Saint’Ynes, Carlos A Menezes, Patricia N Santos, Everardo M Guanabara, Elson J Almeida Jr, Joaquim L Moreira, Maria R Sousa, Frederico A Peret, Liv B Paula, Luiza E Schmaltz, Cleire Pessoni, Leila Katz, Adriana Bione, Antonio C Barbosa Lima, Edilberto A Rocha Filho, Melania M Amorim, Debora Leite, Ivelyne Radaci, Marilia G Martins, Frederico Barroso, Fernando C Oliveira Jr, Denis J Nascimento, Cláudio S Paiva, Moises D Lima, Djacyr M Freire, Roger D Rohloff, Simone M Rodrigues, Sergio M Costa, Lucia C Pfitscher, Adriana G Luz, Daniela Guimaraes, Gustavo Lobato, Marcos Nakamura-Pereira, Eduardo Cordioli, Alessandra Peterossi, Cynthia D Perez, Jose C Peraçoli, Roberto A Costa, Nelson L Maia Filho, Jacinta P Matias, Silvana M Quintana, Elaine C Moises, Fátima A Lotufo, Luiz E Carvalho, Elvira A Zanette, Carla B Andreucci, Márcia M Aquino, Maria H Ohnuma, Rosiane Mattar and Felipe F Campanharo.
